# Dosimetric impact of esophageal motion in esophagus-sparing radiotherapy of spinal metastases

**DOI:** 10.2340/1651-226X.2025.44033

**Published:** 2025-09-22

**Authors:** Anna Mann Nielsen, Laura Ann Rechner, Sebastian Moretto Krog, Vanja Gram, Morten Hiul Suppli, Patrik Sibolt, Ivan Richter Vogelius, Claus P. Behrens, Gitte Fredberg Persson

**Affiliations:** a Department of Radiation Oncology, Copenhagen University Hospital – Herlev and Gentofte, Herlev, Denmark; b Department of Radiation Oncology, Copenhagen University Hospital – Rigshospitalet, Copenhagen, Denmark; c Department of Clinical Medicine, University of Copenhagen, Copenhagen, Denmark; d Department of Health Technology, Technical University of Denmark, Kongens Lyngby, Denmark

**Keywords:** esophagus, organ at risk, spine, bone metastases, palliation

## Abstract

**Background and purpose:**

The phase III trial (NCT05109819) investigates whether esophagus-sparing radiotherapy (RT) reduces dysphagia in patients with spinal metastases. This retrospective simulation study evaluates the dosimetric impact of inter-fractional esophageal motion in this setting.

**Patient/materials and methods:**

Patients receiving daily image-guided RT on high-quality cone beam computed tomography (CBCT)-equipped units between September 2023 and December 2024 were screened. Inclusion required five consecutive CBCTs with vertebrae T1–T10 in view and a visible esophagus. The esophagus was contoured on the planning CT (pCT) and five CBCTs. Standard and esophagus-sparing 25 Gy/5 fraction volumetric-modulated arc therapy plans were simulated for five thoracic targets. In esophagus-sparing plans, the esophageal D0.027cc was limited to 8 Gy equivalent dose in 2 Gy fractions (α/β = 3 Gy), consistent with the ESO-SPARE constraint. CBCT-based esophagus contours were rigidly transferred to the pCT after bony matching. Esophageal metrics (Mean, D0.027cc–D5cc, V8.5 Gy) were extracted from the original plan and compared across CBCT and pCT contours and between plan types. Inter-fraction motion was assessed using 95% Hausdorff Distance (HD). CBCT quality was evaluated in two patients (five targets) using Dice similarity coefficients and interobserver 95% HDs.

**Results:**

Twelve patients with 23 targets were simulated. Inter-fraction motion led to dose constraint violations in 20 of 23 plans, though only small esophageal volumes entered the high-dose region. Dice scores > 0.8 confirmed good esophageal visibility on CBCT. Most 95% HDs fell within interobserver variability, indicating motion was comparable to contouring uncertainty.

**Interpretation:**

Despite constraint violations, high-dose exposure was limited to small esophageal volumes, and overall sparing was preserved.

## Introduction

Palliative radiotherapy (RT) combined with high-dose corticosteroids is the current standard of care for most patients with impending or established metastatic spinal cord compression (MSCC) [1]. The treatment aims to improve patients’ quality of life by alleviating pain and preventing progression of sensory and motor dysfunction. However, in cases where the radiation field includes the esophagus or pharynx, patients may develop acute radiation-induced dysphagia, which can negatively impact their quality of life [2].

The randomized phase III trial ESO-SPARE (NCT05109819) is the first study to investigate if esophagus-sparing Volumetric-Modulated Arc Therapy (VMAT) or Intensity-modulated Radiotherapy (IMRT) can reduce dysphagia in patients with impending or established MSCC in the cervical and thoracic spine [3, 4]. In the esophagus-sparing arm, the dose to the anterior part of the vertebra is compromised to keep the esophagus D_0.027cc_ below8 Gy equivalent dose in 2 Gy fractions (EQD2), assuming an α/β of 3 Gy. This priority results in plans with a horseshoe-shaped dose gradient close to the posterior and lateral parts of the esophagus. Esophagus motion may compromise the effect of the esophagus-sparing strategy if the esophagus moves into the high-dose volume. It can be speculated that this effect is larger for the more mobile distal part of the esophagus. In palliative RT, where treatment typically involves non-adaptive techniques in free breathing, inter-fraction motion is of greatest interest. Due to the limited visibility of the esophagus on cone beam computed tomography (CBCT), previous studies of esophagus motion used barium contrast enhancement, fiducial markers, and CT scans during treatment to estimate inter-fraction motion [5–14]. In September 2023, two treatment units at our institution were upgraded with the HyperSight CBCT platform (Varian, a Siemens Healthineers company), which provides high-quality CBCT images with enhanced esophageal visualization [15].

In this retrospective simulation study, we test the hypothesis that esophageal inter-fraction motion may counteract the effect of the esophagus-sparing intervention in the ESO-SPARE trial. The dosimetric impact of esophageal inter-fraction motion is quantified using consecutive daily high-quality CBCTs.

## Patients/material and methods

### Patient selection

Patients who underwent thoracic palliative RT on HyperSight-equipped units between September 2023 and December 2024 were screened for eligibility. Inclusion criteria were treatment in free breathing, ≥ five fractions, and ≥ five consecutive CBCTs available with any of the thoracic vertebrae T1–T10 in the field of view. Finally, the image quality of the CBCT should allow for manual esophagus segmentation. This study received approval from the Danish data regulatory authorities, and all patients provided informed consent for the use of their data in clinical research.

### Image acquisition

Planning CT scans (pCT) acquired with a 2 mm slice thickness were available in Eclipse (Varian, a Siemens Healthineers Company). For each patient, five consecutive CBCT scans were exported to Eclipse from the Ethos planning system (Varian, a Siemens Healthineers Company).

### Contouring

Simulated spinal targets corresponding to vertebral levels T1–2, T3–4, T5–6, T7–8, and T9–T10 were delineated on the pCT. Targets spanning two vertebral levels were selected, as this represented the most common target volume in the ESO-SPARE trial. According to our local guidelines, the clinical target volume (CTV) for spinal metastases includes the vertebral body, spinal canal, and vertebral arch. The spinous and transverse processes were only included if there was evidence of disease. An isotropic 5 mm planning target volume (PTV) margin was added to the CTV.

A junior physician, supervised by a senior clinical oncologist, delineated the esophagus on the pCT (_P_Esophagus) and each of the five CBCTs (_CBCT_Esophagus_fx1–5_), resulting in six esophageal structures per target level. The esophagus was delineated from at least three slices above to three slices below the PTV.

### Treatment planning

The simulated prescribed dose was 25 Gy in 5 fractions (25 Gy/5 fx) to ≥ 90% of the PTV. This fractionation regimen was the most used in the ESO-SPARE trial and was therefore chosen for the current simulation study. For each included target, two VMAT plans were created: a standard plan where the esophagus was not included in the optimization and an esophagus-sparing plan. In the esophagus-sparing plans, the near max dose D_0.027cc_ (the size of a voxel) was limited to 8.5 Gy (i.e. 1.7 Gy per fraction), the same constraint as used in the ESO-SPARE trial. This corresponds to 8 Gy EQD2 with an α/β of 3 Gy. This limit was applied even if it resulted in compromised PTV and CTV coverage. Supplementary Figure 1 provides an example of target delineation and a comparison between an esophagus-sparing and standard plan.

### Data extraction and analysis

#### CBCT-based esophagus structures

CBCT images were registered to the pCT using bony matching within the PTV, and each of the five esophageal contours (_CBCT_Esophagus_fx1–5_) was rigidly transferred to the pCT. For dosimetry and inter-fraction motion analysis, the esophagus was cropped into sections corresponding to the relevant target levels (3 slices above and below the PTV). Separate analyses were conducted for each level.

For each CBCT-based esophagus structure, the dose was extracted from the original VMAT plan. Importantly, this extracted dose represents the hypothetical dose that the CBCT esophagus would have received had it remained stationary in that position throughout the entire treatment course, meaning that each fraction’s dose constitutes a worst-case scenario. By averaging these fraction-specific doses, we derive an estimate of the delivered dose that accounts for inter-fraction motion in each treatment plan. For example, using the D0.027cc metric, the estimated delivered dose is calculated as follows:

_delivered_D0.027cc = (D0.027cc_fx1_ + D0.027cc_fx2_ + D0.027cc_fx3_ + D0.027cc_fx4_ + D0.027cc_fx5_) / 5.

To address our hypothesis, we aimed to evaluate the number of plans in which protocol-prescribed esophageal constraint was violated due to esophagus inter-fraction motion. Specifically, we assessed the number of patients with a _delivered_D0.027cc > 8.5 Gy and the esophageal volume receiving more than 8.5 Gy (V8.5 Gy in cc).

The dosimetric impact of esophageal inter-fraction motion was further assessed by comparing the delivered and planned dose to selected esophageal volumes – D0.027cc (Gy), D0.5cc (Gy), D1cc (Gy), D2cc (Gy), and esophageal mean dose. The Wilcoxon rank-sum test was used for all dosimetric comparisons. A p value of 0.05 was considered statistically significant.

#### Inter-fraction motion analysis

We used the bidirectional 95% Hausdorff Distance (95% HD) to measure esophagus inter-fraction motion between each CBCT esophagus contour and the pCT esophagus. For every point on the evaluated contour, the distance to the nearest point on the reference contour was computed. The 95% HD is defined as the 95th percentile of these distances, reducing the influence of outliers. The bidirectional 95% HD considers distances in both directions (CBCT to pCT and pCT to CBCT) and averages them, providing a more balanced assessment of shape variations [16]. To facilitate comparison with previous studies, we calculated a 95% Planning Risk Volume (PRV) margin, defined as the margin that would encompass 95% of the observed 95%HD values. HD was calculated in the SlicerRT extension of the 3D Slicer software program (version 5.6.2) [17].

#### Interobserver variation assessment

We assessed interobserver variation in esophagus delineation for two reasons. First, to evaluate the visibility of the esophagus on the HyperSight CBCT, with the assumption that poor visibility leads to greater interobserver variation. Second, to estimate the magnitude of delineation uncertainty, which serves as a reference for evaluating esophageal inter-fraction motion.

From the included patients, two were randomly selected and used to evaluate interobserver variation. In these patients, the esophagus was delineated independently by two physicians on both the pCTs and CBCTs. Structures were compared by Dice similarity coefficient and 95%HD [18].

Statistical analysis was conducted using R, version 4.0.1 (R Foundation for Statistical Computing).

**Figure 1:**
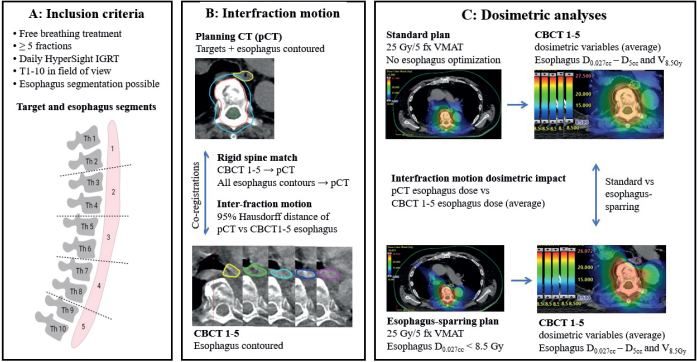
Methods. Panel A shows a schematic overview of the simulated target levels and corresponding esophageal segments. A total of 12 patients and 23 targets were included: five targets each at T1–2, T3–4, T5–6, and T7–8, and three at T9–10. Panels B and C outline the contouring and treatment planning workflow, inter-fraction motion analysis, and dosimetric evaluation.

Figure 1 provides a visual overview of the methods section.

## Results

### Patients

Twelve patients with 23 simulated targets were included in the analysis, distributed as follows: five targets at each level T1–2, T3–4, T5–6, and T7–8 and three targets at the T9–10 level. Baseline characteristics and target distributions are summarized in Supplementary Tables 1 and 2.

### Esophagus inter-fraction motion

**Figure 2:**
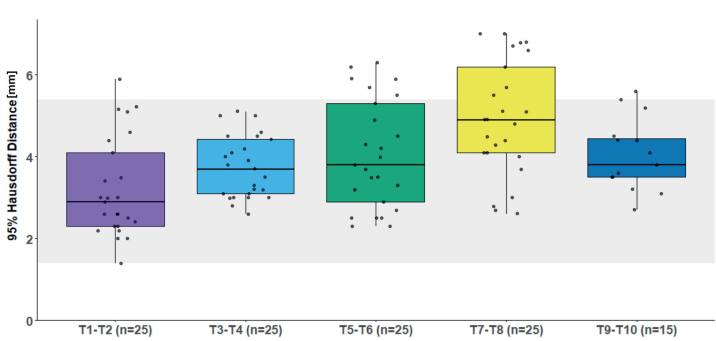
Boxplot of esophagus 95% Hausdorff Distance (HD) across five thoracic metastatic spinal target levels. For spinal levels T1–2, T3–4, T5–6, and T7–8, five targets treated with five fractions each were analyzed. For spinal level T9–10, three targets treated with five fractions each were analyzed. The thick horizontal line represents the median, and the box represents the interquartile range (IQR). The whiskers extend to the smallest and largest values within 1.5 times the IQR from Q1 and Q3. Individual data points are overlaid as black dots. The shaded area represents the range of 95%HD from interobserver variation analysis.

The distribution of 95%HD across all analyzed fractions and spinal levels is presented in Figure 2 and Supplementary Figure 2. Overall, 95%HD mean was 4.0 mm (range [1.4, 7.0] mm). *A 6.4 mm PRV margin is needed to cover 95% of the observed* 95%HD *values.* Most of the observed 95%HD values fell within the range of values seen in the interobserver analysis, suggesting that the variations due to inter-fraction motion were comparable to those arising from differences between observers.

### Dosimetric analysis

**Figure 3:**
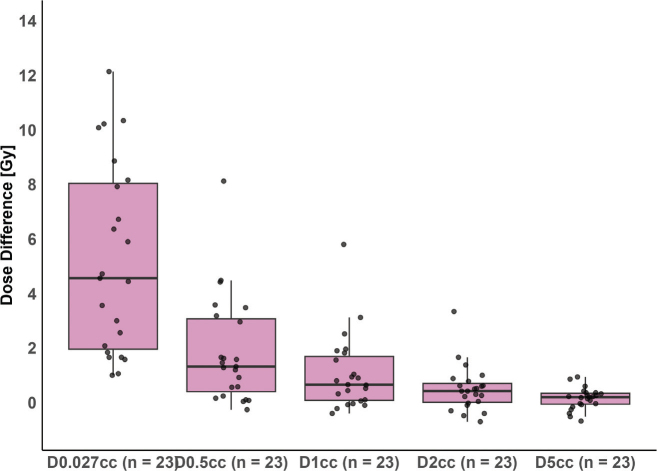
Boxplot of the absolute dose difference between planned and estimated delivered dose for esophagus dose metrics: D0.027cc, D0.5cc, D1cc, D2cc, and D5cc. The thick horizontal line represents the median; the box spans the interquartile range (IQR). Whiskers extend to the smallest and largest values within 1.5 times the IQR from the lower (Q1) and upper (Q3) quartiles. Individual data points are overlaid as black dots.

Dosimetric summary of esophagus-sparing and standard plans is provided in Supplementary Tables 3 and 4. In 20 of 23 plans, the_delivered_D0.027cc exceeded the prescribed constraint of 8.5 Gy. Figure 3 illustrates the distribution of absolute dose differences from planned to delivered dose per fraction for all analyzed dosimetric parameters.

**Figure 4:**
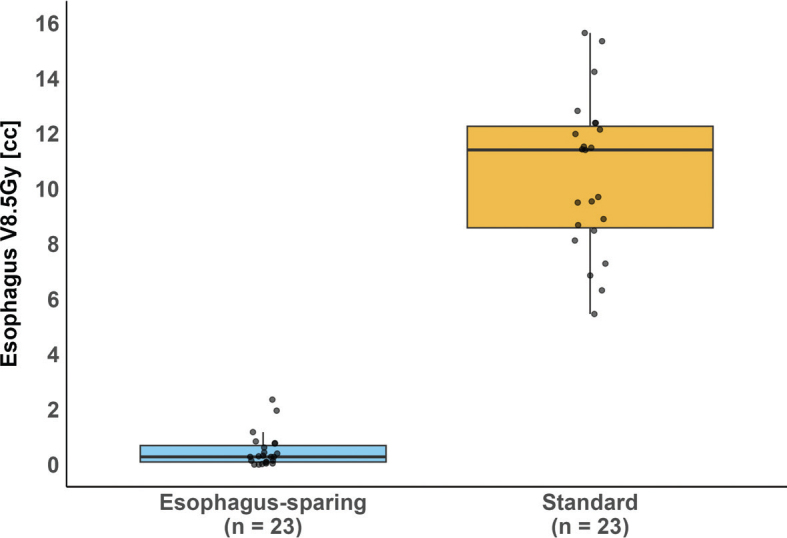
Comparison of CBCT esophagus V8.5 Gy – the volume of the esophagus (cc) receiving more than 8.5 Gy – between esophagus-sparing and standard plans. Each box represents data from 23 simulated 25 Gy/5-fraction plans, where esophagus V8.5 Gy is averaged over five fractions. The thick horizontal line represents the median, while the box indicates the interquartile range (IQR). Whiskers extend to the smallest and largest values within 1.5 times the IQR from Q1 and Q3. Individual datapoints are shown as black dots. The difference in dose metrics was statistically significant according to the Wilcoxon rank-sum test (p < 0.001). CBCT: cone beam computed tomography.

**Figure 5:**
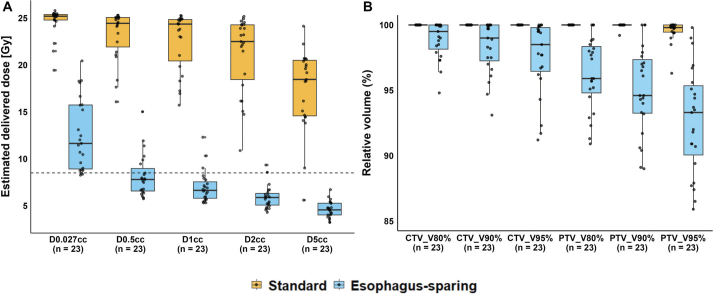
Panel A shows the estimated delivered dose to selected esophageal sub-volumes (D0.027cc, D0.5cc, D1cc, D2cc, and D5cc) for both esophagus-sparing and standard radiotherapy plans. Panel B displays the coverage of the clinical target volume (CTV) and planning target volume (PTV) for the same plan types, illustrating a tradeoff in target coverage. Each box represents data from 23 simulated treatment plans. The thick horizontal line indicates the median; the box spans the interquartile range (IQR), and the whiskers extend to the furthest data points within 1.5 × IQR from the first and third quartiles. Individual data points are shown as black dots. A dashed horizontal line marks 8.5 Gy – the protocol-defined dose constraint for esophageal D0.027cc. For all dose and coverage metrics, the difference between the two plan types was statistically significant (Wilcoxon rank-sum test, p < 0.001).

Notably, changes greater than 4 Gy were observed only in D0.027cc, D0.5cc, and D1cc, suggesting that only a small portion of the esophagus shifted into the high-dose region. This was further supported by the low CBCT esophagus volumes receiving more than 8.5 Gy and visual inspection of dose distributions in cases where constraints were violated, see Figure 4 and Supplementary Figure 3. All evaluated esophageal dose metrics were significantly lower in esophagus-sparing plans compared to standard plans, at the cost of reduced CTV and PTV coverage, see Figure 5 and Supplementary Figures 1 and 3. For the assessment of interobserver variation, the esophagus was delineated in two patients with five targets corresponding to 5 pCTs and 25 CBCTs, see Supplementary Figure 4 and Supplementary Table 5. Although lower than for the pCT, all mean Dice in both locations scores were > 0.8, indicating good visualization of the esophagus on CBCT.

## Discussion and conclusion

In this simulation study of esophagus-sparing RT for complicated spinal metastases, esophagus inter-fraction motion led to the violation of protocol-prescribed esophagus constraint in 20 of 23 simulated plans. However, only a small part of the esophagus moved to the high-dose area, and the overall esophagus sparing-intent was preserved.

RT techniques such as IMRT and VMAT enable precise delivery of doses to tumors while minimizing exposure to organs at risk, making the study of organ motion essential for optimal treatment planning. Esophageal inter-fraction motion has been investigated in two main contexts. First, in the treatment of esophageal cancer, the esophagus is the target, and motion analysis is used to determine appropriate margins for tumor coverage [9–14, 19]. Second, in other thoracic malignancies – primarily lung cancer – the esophagus is considered an organ at risk [5–8, 20]. A summary of studies examining esophageal inter-fraction motion in the latter context are provided in Table 1, with the results of the present study included in the final row.

**Table 1:** Studies reporting esophagus inter-fraction motion in thoracic radiotherapy other than esophagus cancer.

Study	Patients	Imaging	Motion metric	Results	Dosimetric impact
Woodford et al. [5]	NSCLC *n* = 18 (44 fractions analyzed)	Planning Four-Dimensional Computed Tomography (4DCT) Weekly CBCT + barium contrast Soft tissue tumor match	95% HD between pCT esophagus and CBCT esophagus	95% PRV: 15 mm	-
Qui et al. [7]	NSCLC *n* = 35 (595 fractions analyzed)	Planning 4DCT Daily CBCT + barium contrast Bony match	Slice-by-slice center shift between pCT esophagus and CBCT esophagus	**95% PRV:** **Cervical**: −4.2 to 7.1 mm Right–Left (RL) and −4.4 to 5.1 mm (AP) **Proximal thoracic**: −10.3 to 6.0 mm (RL) and −4.3 to 3.8 mm Anterior–Posterior (AP) **Middle thoracic**: −8.7 to 5.5 mm (RL) and −6.4 to 2.8 mm (AP) **Distal thoracic**: −9.1 to 4.7 mm (RL) and −5.8 to 3.3 mm (AP)	-
Gao et al. [6]	NSCLC *n* = 24	Conventional CT in free breathing or breath hold CT week 2 + 5 Bony match	Slice-by-slice center shifts between pCT esophagus and CBCT esophagus	**Upper:** 4.4 mm ± 1.7 **Middle:** 5.5 mm ± 2.0 mm **Lower:** 4.9 ± 2.1 mm	Increased esophageal motion related to increased V60 Gy and increased toxicity.
Pham et al. [20]	NSCLC SBRT *n* = 10 (50 fractions)	Conventional CT in free breathing Daily CBCT Soft tissue tumor match	Esophagus centroid displacement relative to PTV centroid	**R-L:** Median 0.9 mm (range, -5.4 to 3.3 mm) toward the left. **A-P:** 0.7 mm (range, -3.7 to 11.5 mm) posteriorly	Two cases of significant late esophageal toxicity correlated with increased D5cc values due to inter-fraction motion
Wang et al. [8]	Single-fraction spine SBRT *n* = 9 (4 fractions analyzed)	Planning 4DCT. Treatment CBCT. Bony match.	Center of mass shift between pCT esophagus and CBCT esophagus	< 2 mm daily motion	Negligible dosimetric impact
This study	Complicated spinal metastases *n* = 12 (23 targets, 115 fractions)	Daily high-quality CBCT	95% HD between pCT esophagus and CBCT esophagus	95% PRV: 6.4 mm	Increased dose to small esophagus volumes (D0.027cc–D0.5cc)

CBCT: cone beam computed tomography; HD: Hausdorff distance; pCT: planning CT; PRV: Planning Risk Volume; CT: computed tomography; PTV: planning target volume.

Four studies used motion-directed metrics for inter-fraction evaluation [6–8, 20]. Two studies reported a larger shift in the lateral direction, which could impact esophageal dose in our study due to the horseshoe-shaped dose gradient surrounding the esophagus [7, 20]. Qiu et al. found greater motion in the distal esophagus [7].

Esophageal tumor motion during free breathing, as well as inter-fractional motion, has been investigated in esophageal cancer patients using fiducial markers or metal clips for motion tracking [9, 10, 12–14]. All five studies reported greater motion in the distal esophagus, with the largest displacement occurring in the cranio-caudal direction [9, 10, 12–14]. While our results also indicate increased motion in the distal esophagus, we observed a smaller HD95% in the esophageal region corresponding to spinal segments T9–T10 compared to T7–T8. This discrepancy may be attributed to the smaller number of simulated targets in the T9–T10 category (three targets corresponding to 15 fractions versus five targets corresponding to 25 fractions).

Jin et al. reported estimated margins required to compensate for inter-fractional tumor position in the left–right/cranio–caudal/anterior–posterior directions as follows: 4.4 mm/11.3 mm/5.5 mm for the proximal esophagus, 8.9 mm/8.6 mm/9.6 mm for the middle esophagus, and 6.1 mm/12.1 mm/5.7 mm for the distal esophagus [12]. We calculated that a PRV of 6.4 mm would be required to capture 95% of esophageal positional variations during treatment planning. This PRV is smaller than both the margins proposed by Jin et al. and the 15 mm margin proposed by Woodford et al., who also used 95% HD to assess esophageal inter-fraction motion [5, 12]. Notably, 83% of the observed 95%HD values in our study fell within the range of those observed for interobserver variation, indicating that the magnitude of motion was comparable to contouring uncertainty.

The observed smaller inter-fraction motion in our study may be influenced by patient selection, as patients were specifically chosen based on the visibility of the esophagus on CBCT. This selection may have led to the exclusion of patients with significant organ motion, a known factor that can induce imaging artifacts [21].

The 95%HD provides an overall measurement of spatial discrepancy between two contours but does not indicate the direction of motion. Since the focus of this manuscript is the dosimetric impact of esophageal inter-fraction motion – information captured through dosimetric analysis – rather than the precise characterization of esophageal movement, it was deemed reasonable to use a motion metric that does not provide directional information [16]. A qualified PRV margin recommendation requires a motion-directed study assessing both inter- and intra-fraction motion, which lies beyond the scope of this study [6, 19].

Two studies analyzed esophageal inter-fraction motion in patients with clinical data [6, 20]. In a Phase I dose-escalation study of Non-Small Cell Lung Cancer (NSCLC), Gao et al. found that increased esophageal motion was associated with higher esophageal volumes re-ceiving more than 60 Gy and an increased risk of esophageal toxicity [6]. In a retrospective analysis of lung Stereotactic Body Radiation Therapy (SBRT), two pa-tients who developed high-grade late toxicity experienced an unexpected increase in esoph-ageal D5cc dose due to inter-fraction motion [20]. In contrast, Wang et al. reported negligible esophageal motion in four patients treated with single-fraction spinal SBRT, with no significant effect on esophageal dosimetry [8]. In our study, esophageal inter-fraction motion had a negligible dosimetric impact, which does not currently necessitate any changes to our RT delivery technique.

Palliative spinal irradiation is typically delivered using simple RT techniques, with doses ranging from 8 to 30 Gy over 1–10 fractions [1, 22]. In our clinic, spinal metastases with impending or manifest MSCC are treated with VMAT or IMRT in free breathing, and the simulated 25 Gy/5 fx treatment is the most used fractionation schedule [4]. Esophageal toxicity associated with thoracic and cervical spinal VMAT or IMRT was specifically investigated in three prospective studies [2, 4, 23]. A small observation study by Gram et al. found that 11 out of 14 patients treated above T8 experienced esophageal toxicity [2]. Sprave et al. compared three-dimensional conformal RT with IMRT in palliative spinal irradiation and found a lower incidence of dysphagia and esophagitis with IMRT [23]. In the ESO-SPARE phase III trial, 188 patients were randomized to assess whether esophagus-sparing VMAT or IMRT could reduce patient-reported dysphagia compared to standard VMAT/IMRT. This study found a significant reduction in patient-reported dysphagia in the per-protocol analysis, as well as an association between higher esophageal volumes receiving more than 15–25 Gy (EQD2, α/β = 10 Gy) and an increased risk of severe or worse dysphagia [4]. In the current study, the esophagus-sparing plans resulted in only small volumes of the esophagus receiving more than 8.5 Gy, suggesting that the clinical impact of inter-fraction motion is likely negligible.

In studies of esophageal motion, poor visibility of the esophagus on CBCT necessitates the use of additional techniques to enhance visibility [5–7]. The need for supplementary conventional CT during treatment may limit the frequency of evaluations, as daily scanning is both inconvenient and resource-intensive [5]. Other methods, such as fiducial marker placement or barium contrast enhancements, are also burdensome for both patients and clinicians. The HyperSight platform that integrates high-quality CBCT into daily image-guided RT offers a unique opportunity to study daily inter-fraction motion without requiring additional imaging or contrast agents. However, image quality remains a concern, as it may still be insufficient for accurately delineating esophageal structures. While high Dice in both locations scores were observed in our interobserver analysis of CBCT esophagus structures, scores were still lower than those observed when comparing pCT-based esophagus structures. Notably, this study was not designed to answer whether overall HyperSight CBCT esophagus visualization is good enough for accurate segmentation or adaptive RT. Patients were selectively chosen based on visual inspection, and the delineation of CBCT esophagus contours, although not timed, was significantly more time-consuming compared to pCT.

The limitations of this study include the previously mentioned selection bias, a small number of patients, a small number of delineating physicians, and targets for interobserver analysis. Furthermore, some patients had multiple simulated targets, for which esophagus motion and image quality would be inherently correlated. Finally, an optimal analysis would have included patients treated within the ESO-SPARE trial, enabling a direct correlation between esophageal motion and clinical outcomes. However, this was not feasible due to logistical constraints: patients requiring adaptive RT were prioritized for treatment on the HyperSight equipped units, and only four of the patients included in this study were enrolled in the ESO-SPARE clinical trial.

Under the updated ESTRO guidelines, single-fraction RT is now the recommended standard for complicated spinal metastases [1]. Simulation-free online adaptive RT, where targets and organs at risk are delineated on a prior diagnostic CT without requiring a pCT, is emerging as a potential approach in palliative single-fraction RT [24, 25]. When combined with HyperSight CBCT, simulation-free esophagus-sparing RT could be a feasible option. Notably, the Ethos system equipped with HyperSight is already being used for online adaptive treatment of esophageal cancer [26]. In that setting, clinicians have adopted the Thorax Fast protocol for CBCT image acquisition, which is reported to reduce imaging artifacts and enhance image quality – settings that have not yet been tested in our clinic.

## Conclusion

In this simulation study of esophagus-sparing RT for MSCC, esophageal inter-fraction motion led to violations of the protocol-prescribed esophagus constraints in 20 out of 23 plans. However, only a small portion of the esophagus entered the high-dose area, and the overall esophagus-sparing intent was maintained.

## Supplementary Material



## Data Availability

Due to national and European GDPR regulations, the data underlying this study cannot be made publicly available. Anonymized data may be made available upon reasonable request by contacting the corresponding author.
