# Supplementary material for: Dosimetric impact of esophageal motion in esophagus-sparing radiotherapy of spinal metastases
Source: Acta Oncol. 2025 Sep 22;64:44033. doi: 10.2340/1651-226X.2025.44033 (PMC12476049; doi:10.2340/1651-226X.2025.44033)
Supplement: Supplementary file 1 [file AO-64-44033-s1.pdf]

Supplementary material has been published as submitted. It has not been copyedited, or typeset by Acta Oncologica

Supplementary material

Supplementary Figure 1. Example of an esophagus-sparing and standard VMAT plan

Supplementary Table 1. Patient demographics

Supplementary Table 2. Distribution of spinal targets

Supplementary Figure 2. Heatmap of esophagus 95% Hausdorff Distance

Supplementary Table 3. Dosimetry – esophagus-sparing plans

Supplementary Table 4. Dosimetry – standard plans

Supplementary Figure 3. Esophagus-sparing plan with esophagus inter-fraction motion.

Supplementary Figure 4. Inter-observer esophagus delineations on pCT and CBCT

Supplementary Table 5. Inter-observer comparison of pCT and CBCT esophagus structures

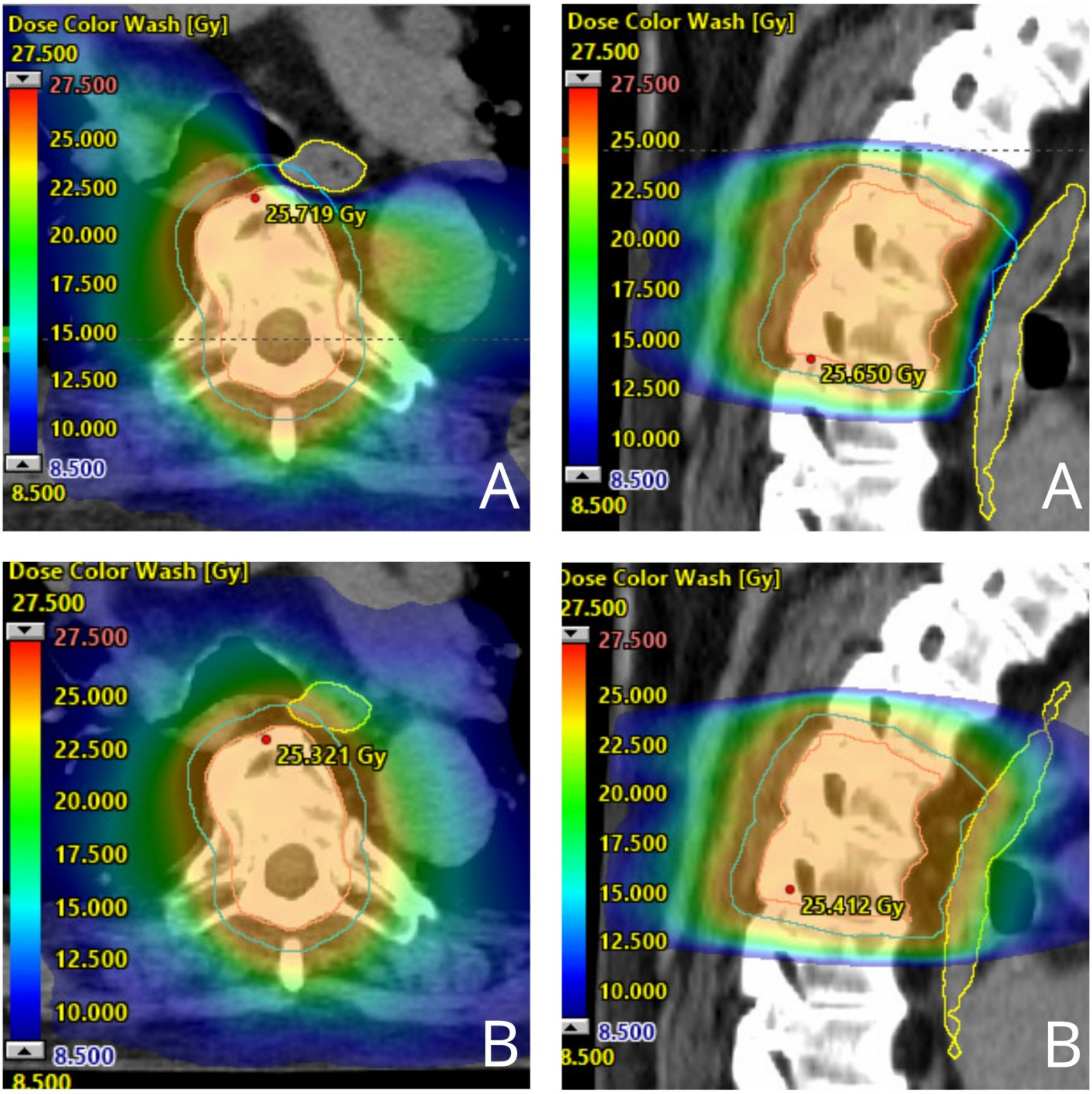

Supplementary Figure 1. *Transversal and axial views of the dose color wash for a 25 Gy in 5 fractions esophagus-sparing plan (Panel A) and a standard plan (Panel B).*

The simulated target includes the T5 and T6 vertebrae. According to our delineation guidelines, the spinous and transverse processes are included in the clinical target volume (CTV) only if there is evidence of disease involvement; therefore, they are not included in the CTV in this figure. The lower threshold of the color wash is set to 8.5 Gy, corresponding to the esophagus dose constraint used in the esophagus-sparing plans in this study.

Supplementary Table 1. Patient demographics.

Age, years, median (range)	74 (52-84)
Male/Female	6/6
**Primary cancer**	
Lung	6
Prostate	2
Breast	2
Other	2
**Radiotherapy intent**	
Palliative lung	5
Metastatic spinal cord compression	6
Palliative clavicle	1

Supplementary Table 2. Distribution of spinal targets and HyperSight CBCT imaging settings.

PT ID	T1-2	T3-4	T5-6	T7-8	T9-10	HyperSight CBCT imaging settings	
1	X					Thorax	125 kV<br>300 mAs
2				X	X	Pelvis	125 kV<br>466 mAs
3		X	X			Pelvis Fast	125 kV<br>529 mAs
4	X	X				Thorax	125 kV<br>325 mAs
5		X	X	X		Thorax	125 kV<br>175 mAs
6	X	X	X			Thorax	125 kV<br>174 mAs
7	X	X	X	X		Pelvis Large Fast	140 kV<br>525 mAs
8					X	Thorax	125 kV<br>175 mAs
9	X		X			Pelvis	125 kV<br>466 mAs
10				X		Pelvis	125 kV<br>915 mAs
11				X		Pelvis Large	140 kV<br>525 mAs
12					X	Pelvis	125 kV<br>466 mAs
Sum	5	5	5	5	3		

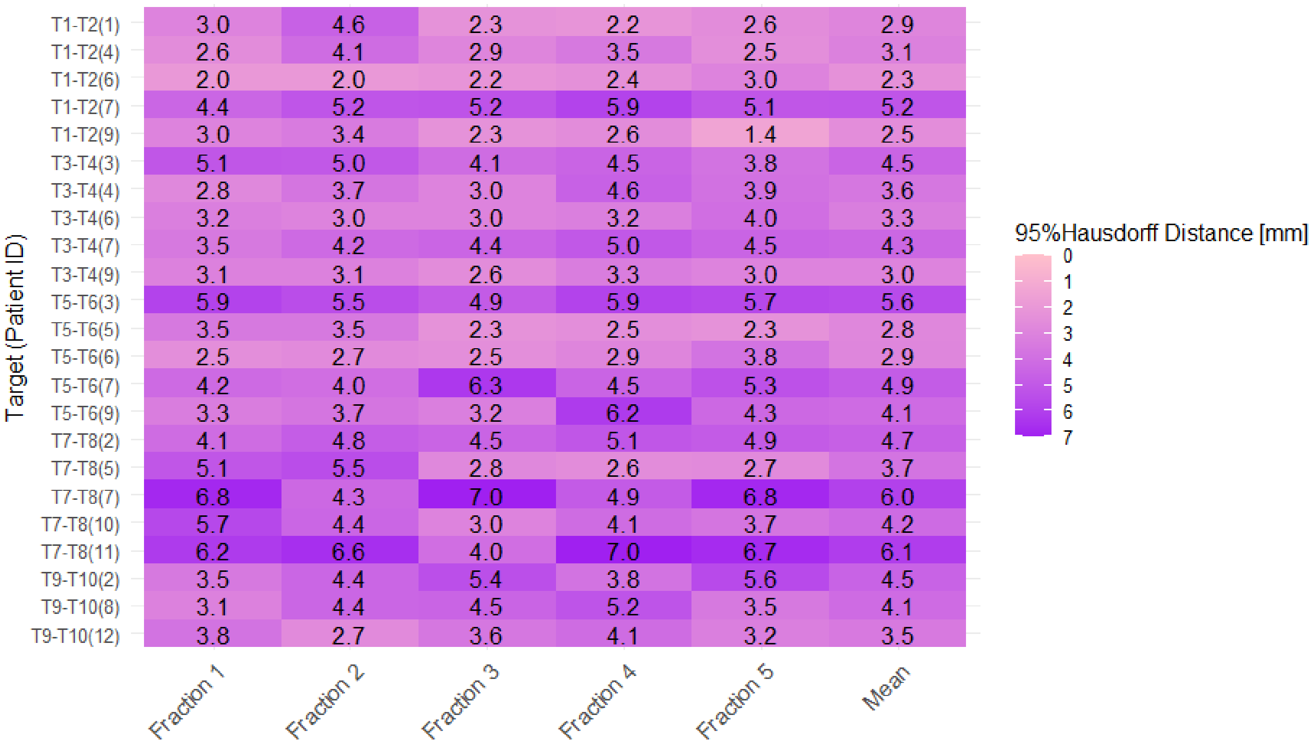

Supplementary Figure 2. *Heatmap of esophagus 95% Hausdorff Distance across five thoracic metastatic spinal cord compression target levels. Each row represents a unique patient-target combination, labeled as Spinal level (ID). The x-axis shows the treatment fractions (1–5) along with the mean value per target. Warmer colors indicate higher Hausdorff Distance values, reflecting greater esophageal motion.*

Supplementary Table 3. Esophageal dose metrics from 25Gy/5Fx esophagus-sparing VMAT plans for complicated spinal metastases.

ID	Target	pMean[a] (Gy)	dMean (Gy) Delivered dose [range]	pD5 (Gy)	dD5 (Gy) Delivered dose [range]	pD2 (Gy)	dD2 (Gy) Delivered dose [range]	pD1 (Gy)	dD1 (Gy) Delivered dose [range]	pD0.5 (Gy)	dD0.5 (Gy) Delivered dose [range]	pD0.027 (Gy)	dD0.027 (Gy) Delivered dose [range]	pV8.5 (cc)	dV8.5 (cc) Volume [range]
1	T1-T2	3.5	3.7 [3.6-3.8]	3.3	3.3 [3.1-3.4]	4.3	4.6 [4.4-4.7]	5.2	5.5 [5.3-5.7]	5.8	6.4 [6.0-6.8]	7.5	9.6 [8.6-11.4]	0	0.1 [0.0-0.2]
2	T7-T8	4.4	4.3 [4.0-5.0]	3.9	3.2 [2.5-4.2]	5.3	4.8 [4.4-6.0]	5.9	5.7 [5.2-7.0]	6.4	6.5 [5.9-8.5]	8.0	9.0 [7.4-14.4]	0	0.1 [0.0-0.5]
2	T9-T10	4.7	4.9 [4.3-5.5]	3.7	3.7 [3.3-4.1]	5.2	5.4 [4.7-5.8]	5.8	6.5 [5.4-7.9]	6.2	7.5 [5.9-9.1]	7.3	12.0 [7.9-14.3]	0	0.3 [0.0-0.6]
3	T3-T4	4.9	5.2 [4.7-5.9]	4.6	4.8 [4.5-5.0]	5.6	6.2 [5.6-6.8]	6.2	7.8 [6.3-9.5]	6.7	10.3 [7.0-14.5]	7.9	18.1 [11.3-24]	0	0.8 [0.2-1.2]
3	T5-T6	4.6	5.0 [4.6-5.5]	3.8	4.7 [4.4-5.0]	5.2	6 [5.5-6.4]	6.9	7.3 [6.2-9.4]	6.4	7.9 [4.4-10.5]	7.8	16.7 [11.2-23.9]	0	0.6 [0.2-1.1]
4	T1-T2	4.0	4.3 [3.9-4.6]	4.6	4.7 [4.6-5.0]	5.5	5.9 [5.5-6.3]	6.0	6.9 [6.0-7.9]	6.7	8.3 [6.7-10.2]	8.5	15.2 [9.3-19.5]	0	0.4 [0.1-0.8]
4	T3-T4	4.4	4.8 [4.5-5.1]	5.0	5.4 [5.0-5.7]	5.7	6.7 [5.9-7.8]	6.1	7.9 [6.4-10.2]	6.5	9.5 [6.9-13.2]	7.8	15.7 [12-19.8]	0	0.8 [0.2-1.6]
5	T5-T6	2.1	4.0 [3.9-4.2]	4.4	4.2 [4.1-4.4]	5.1	5.0 [4.8-5.2]	5.4	5.3 [5.0-5.7]	5.7	5.7 [5.4-6.3]	6.7	8.4 [6.7-10.1]	0	0.0 [0.0-0.0]
5	T7-T8	2.4	4.0 [2.1-4.7]	4.9	4.4 [3.2-4.8]	5.6	5.3 [4.2-5.9]	6.0	6.0 [4.8-6.8]	6.2	6.8 [5.2-8.8]	6.9	10.5 [6.7-19.6]	0	0.1 [0.0-0.5]
6	T1-T2	4.6	5.0 [4.8-5.4]	4.6	4.8 [4.6-5.0]	5.6	5.9 [5.6-6.3]	6.0	6.6 [6.2-7.6]	6.3	7.6 [6.8-10.2]	7.2	11.6 [9.0-16.4]	0	0.3 [0.1-0.8]
6	T3-T4	4.1	4.4 [4.1-4.9]	3.8	4 [3.7-4.6]	4.8	5.2 [4.8-6.2]	5.4	5.9 [5.0-7.7]	5.8	6.7 [5.9-9.0]	6.2	8.8 [7.1-12.9]	0	0.1 [0.0-0.7]
6	T5-T6	3.8	4.5 [3.7-6.1]	3.3	3.5 [3.1-3.7]	4.1	4.7 [3.9-5.7]	4.6	6.6 [4.4-12]	5.0	8.5 [4.8-18.8]	6.1	12.5 [6.1-25.2]	0	0.3 [0.0-1.3]
7	T1-T2	5.8	7.0 [6.5-7.3]	6.3	6.7 [6.3-7.0]	6.9	8.6 [7.6-9.0]	7.2	10.3 [8.7-11.1]	7.5	11.9 [10.6-12.9]	8.2	18.3 [17.5-18.9]	0	2.0 [0.9-2.4]
7	T3-T4	5.0	5.8 [5.3-6.0]	5.1	5.7 [5.5-5.8]	5.9	7.3 [6.8-7.6]	6.5	9.0 [8.0-10.1]	6.9	11.4 [9.8-13.7]	8.0	18.3 [15.4-22.9]	0	1.2 [0.8-1.5]
7	T5-T6	4.9	5.3 [4.9-5.5]	5.0	5.3 [4.9-5.5]	5.8	6.7 [5.8-7.0]	6.3	8.2 [6.4-8.9]	6.7	9.9 [7.1-11.3]	7.6	15.8 [10.3-19.3]	0	0.8 [0.1-1.1]
7	T7-T8	5.0	6.4 [5.5-7.5]	5.1	6 [5.1-7.2]	6.0	9.3 [6.7-12.4]	6.5	12.3 [8.4-16.6]	6.9	15.0 [10.6-20.2]	8.3	20.4 [16.8-24]	0	2.4 [1.0-3.9]
8	T9-T10	4.2	4.0 [3.8-4.2]	4.2	3.8 [3.6-4.1]	5.1	4.7 [4.4-5.2]	5.7	5.3 [4.9-5.9]	6.1	5.8 [5.2-6.5]	7.0	8.8 [7.2-11.6]	0	0.0 [0.0-0.1]
9	T1-T2	4.6	4.7 [4.6-4.8]	5.2	5.3 [5.1-5.4]	5.9	5.9 [5.3-6.1]	6.2	6.3 [6.1-6.5]	6.5	6.7 [6.4-7.0]	7.2	8.3 [7.3-9.2]	0	0.0 [0.0-0.0]
9	T3-T4	4.2	4.2 [4.1-4.4]	4.4	4.1 [3.2-4.5]	5.1	5.1 [4.9-5.4]	5.6	5.7 [5.3-6.0]	6.0	6.2 [5.7-6.9]	7.1	8.8 [8.1-10.4]	0	0.0 [0.0-0.2]
9	T5-T6	4.2	4.2 [4.0-4.4]	4.1	4.0 [3.8-4.2]	5	4.3 [2.0-5.2]	5.5	5.4 [5.0-5.8]	5.9	6.0 [5.6-6.6]	6.9	8.5 [6.9-10.0]	0	0.0 [0.0-0.1]
10	T7-T8	4.1	5.3 [4.7-6.1]	5.1	5.4 [4.7-6.4]	5.8	6.4 [5.3-7.8]	6.1	7.1 [5.7-8.7]	6.3	7.9 [6.0-9.5]	6.9	11.5 [7.7-14.3]	0	0.4 [0.0-1.2]
11	T7-T8	4.7	5.2 [4.9-5.4]	4.2	4.6 [4.4-4.7]	5.2	5.7 [5.4-5.9]	5.8	6.7 [6.1-7.2]	6.3	8.0 [6.7-8.9]	7.2	13.1 [8.5-15.1]	0	0.3 [0.0-0.6]
12	T9-T10	4.9	5.3 [5.0-5.6]	4.3	4.6 [4-5.2]	5.6	6.1 [5.7-6.8]	6.2	7.0 [6.5-7.8]	6.6	7.8 [7.2-8.7]	7.7	10.7 [9.2-11.8]	0	0.3 [0.1-0.6]

[a]Planned dose values (e.g., pMean [Gy], pDXcc [Gy]) represent the dose calculated for the target areas during treatment planning.

[b]Delivered dose values (e.g., dMean [Gy], dDXcc [Gy]) represent the estimated delivered dose while accounting for esophagus inter-fraction motion . Calculation example using D0.027cc: dD0.027cc = ($D0.027cc_{fx1}$ + $D0.027cc_{fx2}$ + $D0.027cc_{fx3}$ + $D0.027cc_{fx4}$ + $D0.027cc_{fx5}$) / 5.

Supplementary Table 4. Esophageal dose metrics from 25Gy/5Fx standard VMAT plans for metastatic spinal cord compression**.**

ID	Target	pMean[a] (Gy)	dMean (Gy)[b] Delivered dose [range]	pD5 (Gy)	dD5 (Gy) Delivered dose [range]	pD2 (Gy)	dD2 (Gy) Delivered dose [range]	pD1 (Gy)	dD1 (Gy) Delivered dose [range]	pD0.5 (Gy)	dD0.5 (Gy) Delivered dose [range]	pD0.027 (Gy)	dD0.027 (Gy) Delivered dose [range]	pV8.5 (cc)	dV8.5 (cc) Volume [range]
1	T1-T2	18.1	21.0 [17.8-25.3]	18.7	14.1 [8.7-18.3]	24.2	22 [18.7-24.3]	25	24.7 [24.2-25.1]	25.3	24.2 [20.1-25.3]	25.8	25.8 [25.8-25.8]	8.7	8.1 [7.1-8.9]
2	T7-T8	23.4	25.0 [22.4-33.3]	7.3	5.6 [4.6-7.0]	15.9	10.9 [5.9-17.0]	20	18.8 [17.8-21.3]	22.1	21.1 [20.3-23.1]	25.2	25.1 [24.9-25.3]	15.9	15.6 [15-16.9]
2	T9-T10	22.1	22.3 [21.8-22.9]	17.8	20.4 [17.7-23]	24.7	24.7 [24.4-24.8]	25	25.0 [24.8-25.0]	25.1	25.1 [25-25.1]	25.3	25.3 [25.2-25.3]	5.9	6.3 [5.5-7.8]
3	T3-T4	14.5	14.1 [13.5-14.8]	13.9	14.7 [13.9-15.2]	17.1	17.9 [17.0-18.7]	19	19.8 [18.8-21.1]	20.8	21.4 [20.2-22.7]	24.1	24.1 [23.6-24.6]	8.1	9.7 [8.5-11.1]
3	T5-T6	11.5	11.5 [11.3-11.8]	10.4	13.8 [12-19.5]	13.9	15.1 [14.0-17.2]	15.1	15.7 [15.4-16.1]	16.2	16.1 [12.7-17.4]	18.6	22.3 [20.0-24.5]	5.1	9.5 [8.6-10.4]
4	T1-T2	16.9	16.8 [16.2-17.5]	19.0	20.6 [19.9-21.2]	24.2	24.3 [23.8-24.7]	24.9	24.9 [24.7-25.1]	25.1	25.1 [24.9-25.2]	25.4	25.4 [25.3-25.4]	11.9	12.4 [9.8-14.6]
4	T3-T4	15.9	15.1 [10.6-16.4]	19.6	19.9 [13.7-22.3]	24.0	23.9 [22.5-24.6]	24.8	24.8 [24.5-25]	25.1	25.1 [25.0-25.2]	25.4	25.5 [25.4-25.5]	13.2	15.3 [8.9-19.5]
5	T5-T6	17.5	17.4 [17.0-17.9]	21.1	20.6 [19.9-21.2]	24.1	24.3 [23.8-24.7]	24.6	24.9 [24.7-25.1]	24.8	25.1 [24.9-25.2]	25.2	25.4 [25.3-25.4]	12.4	12.4 [9.8-14.6]
5	T7-T8	16.5	16.6 [16.1-17.5]	10.1	18.5 [17.8-19.5]	19.1	21.5 [20.5-22.9]	21.7	23.0 [22.2-24]	23.3	23.4 [22.1-24.4]	24.6	24.7 [24.5-24.9]	15.2	14.2 [12.7-16.2]
6	T1-T2	17.3	18.5 [18.1-18.9]	18.1	20 [19.3-21.4]	23.9	24.2 [24.0-24.5]	24.7	24.8 [24.7-24.9]	25.0	25.1 [25.0-25.1]	25.3	25.3 [25.3-25.3]	8.2	8.7 [8.1-9.7]
6	T3-T4	10.4	10.7 [9.9-11.6]	8.0	9.0 [7.5-11.3]	14.1	14.7 [13.0-17.6]	10.5	18.3 [16.6-20.9]	20.6	20.9 [19.3-23]	25.4	25.4 [25.0-26.0]	4.7	5.5 [4.4-7.2]
6	T5-T6	17.2	17.9 [17.1-18.7]	14.2	16.2 [11.9-17.9]	20.9	22.2 [20.2-23.7]	22.9	23.7 [22.2-24.7]	23.9	24.4 [23.3-25]	25.1	25.2 [24.9-25.3]	6.1	6.9 [5.5-7.4]
7	T1-T2	19.6	20.0 [19.6-20.8]	23.9	24.1 [23.2-24.6]	25.1	25.2 [25.1-25.2]	25.3	25.3 [25.2-25.3]	25.3	25.3 [25.3-25.3]	25.5	25.5 [25.4-25.5]	11.2	11.4 [10.3-12.1]
7	T3-T4	18.1	18.5 [18.2-18.8]	21.3	22.2 [21.9-23.0]	24.8	24.9 [24.8-25.1]	25.2	25.2 [25.2-25.3]	25.3	25.3 [25.3-25.3]	25.5	25.5 [25.5-25.5]	10.7	12.1 [11.5-13.3]
7	T5-T6	17.1	16.6 [15.6-17.2]	18.8	19.2 [17.7-20.0]	23.1	23.2 [21.6-23.9]	24.4	24.4 [23.4-24.8]	24.9	24.9 [24.5-25.1]	25.4	25.2 [24.5-25.4]	10.1	11.5 [10-12.7]
7	T7-T8	16.6	17.7 [17.3-18.4]	17.4	19.6 [18.5-21.3]	21	23.2 [22.4-24.5]	22.9	24.4 [23.9-25]	23.8	24.8 [24.6-25.2]	25.0	25.4 [25.2-25.5]	9.7	12.8 [11.1-14.5]
8	T9-T10	18.4	18.1 [17.7-19.0]	19.9	18.3 [16.8-20.3]	23.2	22.4 [21.6-23.4]	24	23.0 [21.1-23.8]	24.3	24.1 [23.8-24.4]	24.8	24.8 [24.6-24.9]	11.0	9.5 [8.2-11.3]
9	T1-T2	16.0	16.2 [15.7-16.7]	20.8	20.7 [19.9-21.4]	23.9	23.9 [23.8-24]	24.3	24.4 [24.3-24.4]	24.6	24.6 [24.5-24.6]	24.9	24.9 [24.8-24.9]	11.6	12.0 [10.8-13.2]
9	T3-T4	14.0	12.9 [9.5-14.1]	15.1	15.1 [14.1-16.1]	20.1	19 [18.1-20.1]	22.4	21.0 [20.1-22.0]	23.7	22.5 [21.6-23.2]	25.1	24.8 [24.2-25.1]	9.7	11.4 [10-13.5]
9	T5-T6	14.3	13.7 [13.2-14.2]	15.0	14.4 [14.0-14.8]	16.9	16.1 [15.3-16.6]	17.9	17 [16.0-17.5]	18.6	17.6 [16.5-18.2]	20.8	19.4 [17.9-20.5]	9.3	8.9 [8.1-9.4]
10	T7-T8	13.4	13.9 [12.9-14.9]	14.2	14.5 [13.5-15.7]	15.6	16.2 [14.8-17.5]	16.3	17.2 [15.6-18.7]	17.2	18.4 [16.2-20.1]	20.5	21.5 [17.4-23.9]	11.0	11.5 [9.3-13.9]
11	T7-T8	18.5	19.2 [18.5-19.7]	17.1	18.2 [17.1-19.3]	22.2	22.5 [21.3-23.4]	23.8	23.8 [23.1-24.4]	24.3	24.4 [24.0-24.8]	25.2	25.2 [25.1-25.3]	6.1	8.5 [8.0-9.3]
12	T9-T10	21.3	21.0 [20.3-21.3]	21.2	20.7 [17.8-22.4]	24.6	24.5 [24.3-24.7]	24.9	24.9 [24.8-24.9]	25.0	25.0 [24.9-25.1]	25.2	25.2 [25.2-25.2]	6.8	7.3 [5.9-8.9]

[a]Planned dose values (e.g., pMean [Gy], pDXcc [Gy]) represent the dose calculated for the target areas during treatment planning.

[b]Delivered dose values (e.g., dMean [Gy], dDXcc [Gy]) represent the estimated delivered dose while accounting for esophagus inter-fraction motion . Calculation example using D0.027cc: dD0.027cc = $(D0.027cc_{fx1} + D0.027cc_{fx2} + D0.027cc_{fx3} + D0.027cc_{fx4} + D0.027cc_{fx5}) / 5$.

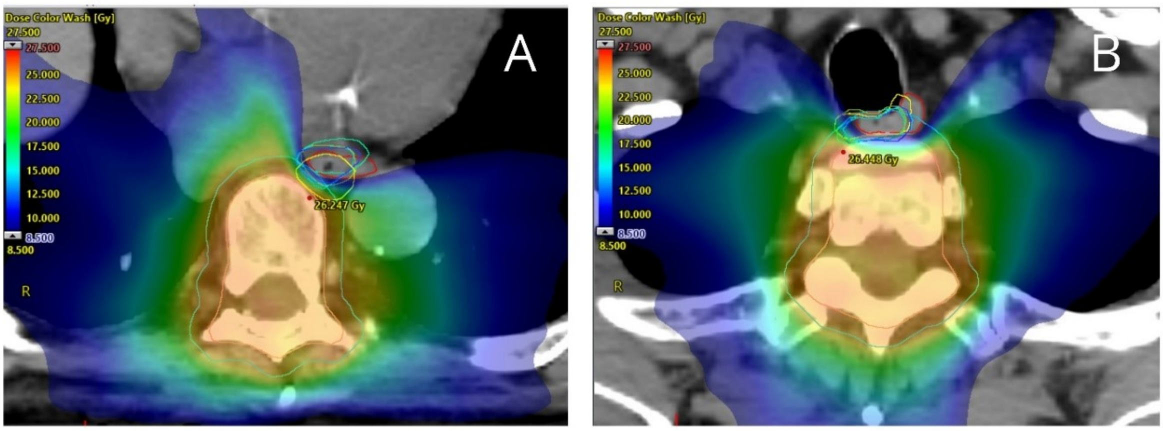

Supplementary Figure 3. *Example of an esophagus-sparing VMAT plan at the level of the T8 vertebra (A) and T1 vertebra (B). The red esophagus contour was used for treatment planning, while the remaining contours represent the esophagus segmented in fractions 1-5. Esophagus inter-fraction motion caused a small portion of the esophagus to move into the high-dose area, violating the esophagus constraint prescribed by the protocol, which is represented by the lower boundary of the dose color wash setting.*

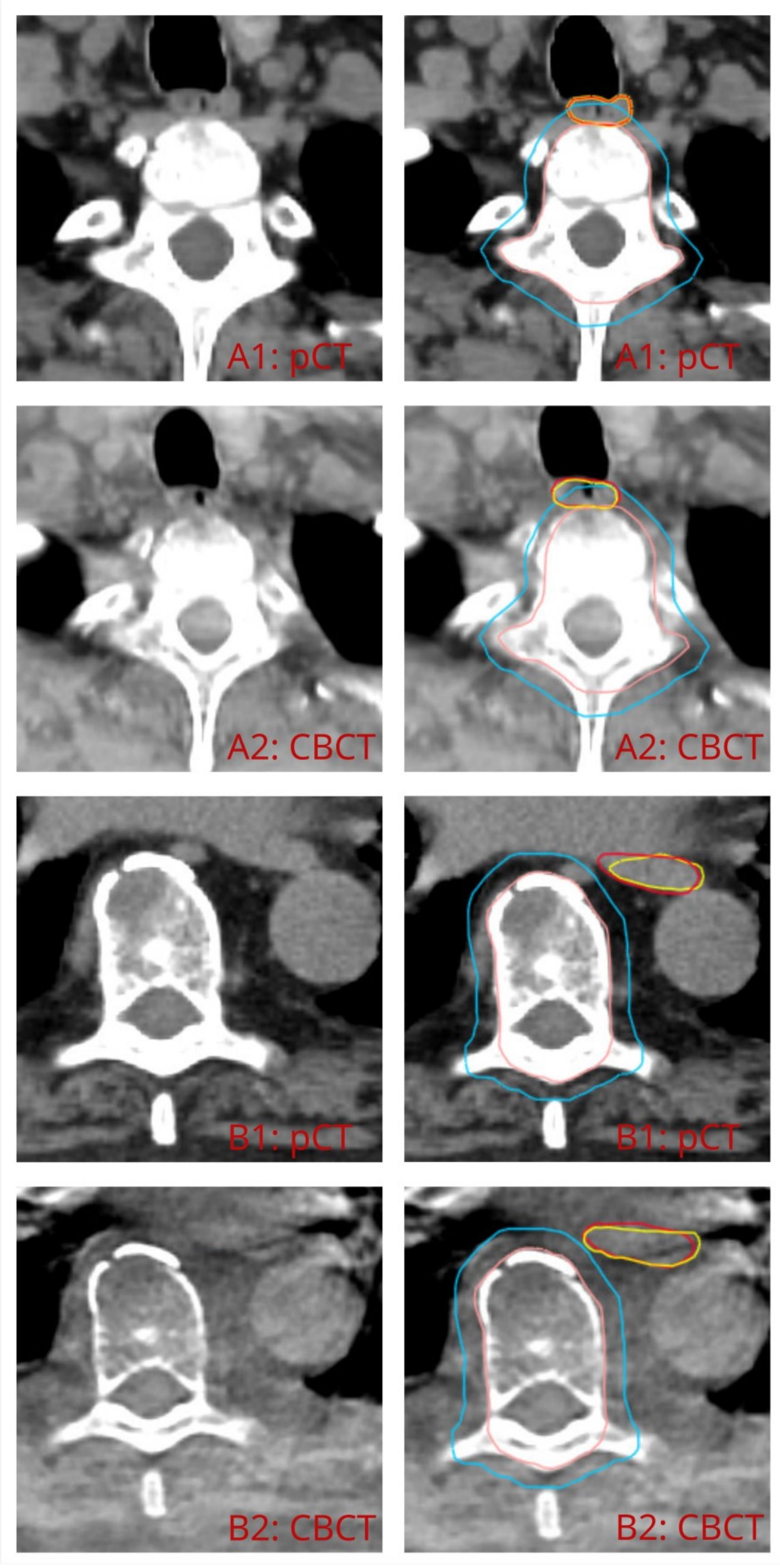

Supplementary Figure 4. *Example of esophagus delineation by two observers. The yellow contour represents the esophagus delineated by Observer 1, and the red contour represents the delineation by Observer 2. Panels A1 and A2 show axial views of the planning CT (pCT) and cone-beam CT (CBCT) images at the level of the T1 vertebra. Panels B1 and B2 show axial views of the pCT and CBCT images at the level of the T8 vertebra. Examples are shown with and without delineations for comparison.*

**Supplementary Table 5.** Inter-observer comparison of CBCT based esophagus structures.		
	Patient 5	Patient 7
	Dice similarity coefficient<br>Mean [range]	
Esophagus delineated on pCT		
pEsophagus T1-2	-	1.0
pEsophagus T3-4	-	1.0
pEsophagus T5-6	0.91	0.99
pEsophagus T7-8	0.91	0.99
Esophagus delineated on CBCT		
$_{CBCT}$Esophagus T1-2	-	0.89 [0.87,0.92]
$_{CBCT}$Esophagus T3-4	-	0.90 [0.89,0.92]
$_{CBCT}$Esophagus T5-6	0.85 [0.83,0.88]	0.86 [0.76,0.91]
$_{CBCT}$Esophagus T7-8	0.83 [0.76,0.87]	0.81 [0.66,0.89]
	95% Hausdorff distance/[mm]<br>Mean [range]	
$_{CBCT}$Esophagus T1-2	-	1.9 [1.4,2.4]
$_{CBCT}$Esophagus T3-4	-	1.5 [1.4,1.7]
$_{CBCT}$Esophagus T5-6	3.12 [2.3,3.8]	2.3 [1.4,4.5]
$_{CBCT}$Esophagus T7-8	3.14 [2.0,3.9]	3.12 [1.4,5.4]

Abbreviations: CBCT refers to cone beam computed tomography. pCT refers to planning computed tomography.
